# Zebrafish Model of Severe Combined Immunodeficiency (SCID) Due to JAK3 Mutation

**DOI:** 10.3390/biom12101521

**Published:** 2022-10-20

**Authors:** Faiza Basheer, Effie Lee, Clifford Liongue, Alister C. Ward

**Affiliations:** 1 School of Medicine, Deakin University, Waurn Ponds, Geelong, VIC 3216, Australia; 2 Institute of Mental and Physical Health and Clinical Translation, Deakin University, Waurn Ponds, Geelong, VIC 3216, Australia; 3 Gribbles Veterinary Pathology, Glenside, SA 5065, Australia

**Keywords:** JAK3, immunodeficiency, SCID, lymphoid cells, leukemia, zebrafish

## Abstract

JAK3 is principally activated by members of the interleukin-2 receptor family and plays an essential role in lymphoid development, with inactivating JAK3 mutations causing autosomal-recessive severe combined immunodeficiency (SCID). This study aimed to generate an equivalent zebrafish model of SCID and to characterize the model across the life-course. Genome editing of zebrafish *jak3* created mutants similar to those observed in human SCID. Homozygous *jak3* mutants showed reduced embryonic T lymphopoiesis that continued through the larval stage and into adulthood, with B cell maturation and adult NK cells also reduced and neutrophils impacted. Mutant fish were susceptible to lymphoid leukemia. This model has many of the hallmarks of human SCID resulting from inactivating JAK3 mutations and will be useful for a variety of pre-clinical applications.

## 1. Introduction

Cytokines and their downstream signaling components play major roles in regulating blood and immune cell development and function [1,2]. Janus kinases (JAKs) are receptor-associated tyrosine kinases that are pivotal in cytokine receptor-mediated signal transduction, such that cytokine binding triggers their activation thereby facilitating stimulation of downstream pathways controlling critical cell processes such as proliferation, survival and differentiation [3]. Disruption of JAKs has been associated with numerous blood and immune diseases [4].

The mammalian JAK family is composed of four members, with JAK3 unique in having restricted expression within the hematopoietic compartment, particularly lymphoid cells [5]. JAK3 predominantly associates with the interleukin-2 receptor gamma common (IL-2Rγc) signaling chain, which is shared by multiple cytokine receptors that regulate lymphoid differentiation and function, specifically IL-2, IL-4, IL-7, IL-9, IL-15 and IL-21 [6,7,8,9]. Activating JAK3 mutations have been associated with a variety of lymphoid malignancies [10,11,12]. In contrast, inactivating JAK3 mutations cause autosomal-recessive severe combined immunodeficiency (SCID) in humans, characterized by a lack of T and Natural Killer (NK) cells and non-functional B cells, due to loss of function for the relevant cytokine receptors [13,14,15].

Zebrafish have become a pivotal platform for the study of blood and immune cells [16], and share high conservation of cytokine receptor signaling components, such as equivalent IL-2Rγc [17] and JAK3 [3] proteins. It therefore represents a highly suitable organism in which to model relevant human diseases, including immunodeficiency [18,19,20] and leukemia [21,22]. This study describes the generation of zebrafish harboring *jak3* mutations similar to those seen in human SCID and the characterization of the blood and immune cells within these mutants throughout the life-course.

## 2. Materials and Methods

### 2.1. Zebrafish Husbandry

Zebrafish were maintained using standard husbandry practices [23], following National guidelines of animal use and care. Embryos were obtained by manual spawning of adult fish and were subsequently maintained at 28.5 °C in a Petri dish containing aquarium water supplemented with 0.003% 1-phenyl-2-thio-urea (PTU) at 8 h post fertilization (hpf) to inhibit pigmentation. All studies involving animals were performed under approval from the Deakin University Animal Welfare Committee.

### 2.2. Genetic Manipulation and Analysis

Wild-type one-cell stage embryos were microinjected with 12.5 ng/µL guide RNA (gRNA) targeting exon 13 of zebrafish *jak3* along with 100 ng/µL Cas9 mRNA (Sigma), as previously described [21]. The microinjected embryos were raised to adulthood, outcrossed with wild-type fish, and potential mutant founders identified using high resolution melt (HRM) analysis [24] with *jak3* primers (5′-TTATCCATGTGAATAAATGTTTAATCTTC, 5′-CTAATGCCATACACCAAAAGAAGG). Mutations were confirmed by sequencing with alternative *jak3* primers (5′-GAGGCTAATAATTCTGACTTTAACTG, 5′-AGTTACTTACTTTTGGATTTGTGCACAC). These founders were out-crossed for two generations followed by in-crossing to generate wild-type, heterozygous and homozygous mutant *jak3* fish.

### 2.3. Whole-Mount In Situ Hybridization (WISH)

Embryos were collected at specific time points, anesthetized with 0.4 mg/mL benzocaine and fixed in 4% (*w*/*v*) paraformaldehyde (PFA) in phosphate-buffered saline (PBS) before storing them at 4 °C. Embryos were subsequently hybridized using digoxygenin (DIG)-labelled RNA probes as described previously [25]. Images were taken using an Olympus MVX10 monozoom microscope with a 1× MVXPlan Apochromat lens (NA = 0.25) and DP74 camera and quantitation performed by manual counting of dispersed individual cells or by measuring the area of the thymus utilizing CellSens Dimensions 1.6 software (Olympus, Notting Hill, Australia). Data from approximately 30 embryos were collected and analyzed for significance using a Student *t*-test with data tested for normality and Welch’s correction performed where necessary.

### 2.4. RT-PCR and qRT^2^-PCR Analysis

Total RNA from zebrafish larvae and adult kidney was extracted using RNeasy Mini Kit (Qiagen, Hilden, Germany) according to the manufacturer’s protocol. cDNA was synthesized using an QuantiTect Reverse Transcription Kit (Qiagen) and was subjected to semi-quantitative reverse-transcription polymerase chain reaction (RT-PCR) using GoTaq (Promega, Madison, WI, USA) with primers for immunoglobulin variable heavy chains *igvh1* (5′-GATGGACGTGTTACAATTTGG, 5′-CCTCCTCAGACTCTGTGGTGA) and *igvh4* (5′-CAAGATGAAGAATGCTCTCTG, 5′-TGTCAAAGTATGGAGTCGA), *jak3* (5′-AACTCAGAGACCACCTTCAGCA, 5′-ACTTTCACTCCAGATGAGGTCC), and T cell receptor beta (TCRβ) variable chains *vb1.5*/*17.5* (5′-AATGGACAGCTTGATAGAACTGAAC, 5′-TGCTTATTCAACCGAACAGAAACATTC), *vb12* (5′-CAGACACCGTGCTTCAGTCGAG, 5′-ACGTTTCATGGCAGTGTTACCTG) and *vb14.5* (5′-GAATCCAATGTGACGTTAACATGC, 5′-CATGATCATAAGGACCACTACAG) or quantitative real-time reverse-transcription PCR (qRT^2^-PCR) using a Sensifast lo-ROX kit (Meridian Bioscience, Cincinnati, OH, USA) with *actb* (5′-TGGCATCACACCTTCTAC, 5′-AGACCATCACCAGAGTCC), *cd4* (5′-TCTTGCTTGTTGCATTCGCC, 5′-TCCCTTTGGCTGTTTGTTATTGT), *cd8* (5′-ACTCTTCTTCGGAGAGGTGAC, 5-ACAGGCTTCAGTGTTGTTTGAA), *cd79a* (5′-GCGAGGGTGTGAAAAACAGT, 5-CCCTTTCTGTCTTCCTGTCCA), *hbba1* (5′-ATGGTTGAGTGGACAGATGC, 5′-TACACGATCAGACATCTGGATAG), *ighd* (5′-TCCTTGCACCATTCTGCTCC, 5′-AAAACCCGACACCAGACACA, *ighm* (5′-CCGAATACAGTGCCACAAGC, 5′-TCTCCCTGCTATCTTTCCGC), *lmo2* (5′-TTACCTCAGACTGTTTGGTCAGG, 5′-CGCACACGCATGGTCATTTC), *mpeg1.1* (5′-CACCTGCTGATGCTCTGCTG, 5′-CCAGACCTCCCAACACCAAC), *mpo* (5′-CTGCGGGACCTTACTAATGATG, 5′-CCTGGATATGGTCCAAGGTGTC), *nccrp1* (5′-TCAGCACAGGTGGTTCACTCTA, 5′-GGCTTTCCTCATACCAGTCTTC), *nklc* (5′-TCATCTCCTCTGCTTGTGCTG, 5′-TTCCTCCTTATTTGCTGTATTGG), *nkld* (5′-TGGTGAAATCCCAACAGAGCA, 5′-TTTCATCCTGAGTTGCACCA), *pax5* (5′-AAGGCAGTTACTCCACACCC, 5′-ACCGTACTCCTGCTGAAACAC), *rag1* (5′-GGATGTGAAGTATGTGTGTTTGA, 5′-TGGAACCCAGGGAGAAGC), *tcra* (5′-ACTGAAGTGAAGCCGAAT, 5′-CGTTAGCTCATCCACGCT), and *tcrb* (5′-AGTTGCAGGTGGATATGACCG, 5′-ATGACAAGGCCATACAGTCCG). Data were normalized to *actb* and fold change was calculated using the ΔΔCt method [26].

### 2.5. Ex Vivo Analyses

Cytospin preparations of adult blood were stained with Giemsa (Sigma, St. Louis, MO, USA), imaged using a Leica DM E microscope with a 100× oil objective (NA = 1.25) and an Olympus SC50 camera and differential counts performed. Zebrafish that developed lymphocytic leukemia were fixed in formalin, followed by paraffin embedding, sectioning and staining with Hematoxylin and Eosin and imaged using an Olympus BX46 clinical microscope with a UPLFLN (PH) Plan Semi Apochromat (OFN 26.5) and DP22 camera, utilizing Olympus CellSens Entry 3.2 (Build 23706) software.

### 2.6. Survival Analysis

Adult fish were monitored by regular visual inspection with relative survival displayed as a Kaplan–Meier curve and statistical significance determined using a log-rank (Mantel-Cox) test.

## 3. Results

### 3.1. Generation of SCID-Related Jak3 Mutant Zebrafish

**Figure 1:**
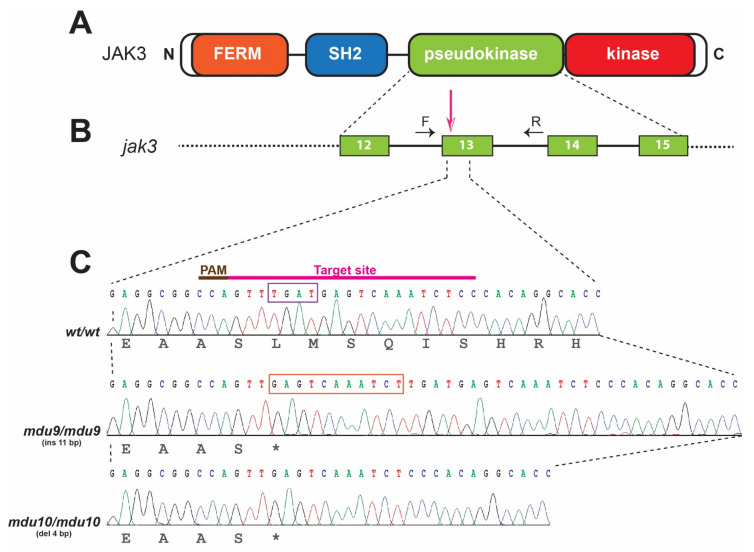
Generation of zebrafish carrying Jak3 mutations resembling those found in autosomal-recessive SCID. (**A**). Schematic representation of the JAK3 protein showing FERM (orange), SH2 (blue), pseudokinase (green) and kinase (red) domains. (**B**). Intron-exon structure of the zebrafish *jak3* gene region encoding the pseudokinase domain, with the area targeted denoted with the pink arrow and the genotyping primers indicated by black arrows (F: forward, R: reverse). Exons are shown as numbered boxes and introns as solid lines. (**C**). Nucleotide sequence of zebrafish homozygous for wild-type (*wt*) and mutant (*mdu9* and *mdu10*) alleles of *jak3*, with their protein translations displayed below in black text and the CRISPR/Cas9 target site shown above. The *mdu9* allele represents an 11 bp insertion (orange box) and the *mdu10* allele a 4 bp deletion (purple box), both resulting in frameshifts that introduce a stop codon at the equivalent location within the pseudokinase domain.

A variety of inactivating mutations of human *JAK3* have been identified in autosomal recessive SCID patients, with the pseudokinase domain (PKD) being a hot spot for such mutations [13,27]. To generate similar mutations in zebrafish Jak3, CRISPR/Cas9-mediated genome editing was employed to target exon 13 of the zebrafish *jak3* gene that encodes the PKD (Figure 1B). One-cell stage zebrafish embryos were injected with Cas9 mRNA and in vitro transcribed gRNA specific for this exon (Figure 1C). These were raised to adulthood and their progeny screened by HRM analysis followed by sequencing. This identified two mutant alleles, *mdu9* and *mdu10*, which carried an 11 bp insertion and a 4 bp deletion at the target site, respectively. Both of these mutations introduced a premature stop codon leading to a Jak3 protein truncated in the PKD at the same amino acid (Figure 1C). Another allele, *mdu11*, carried a 2 bp deletion, leading to a frameshift at the same site. Founder fish carrying these alleles were out-crossed twice to dilute any potential off-targeting, with F2 heterozygous mutants in-crossed to generate F3 wild-type, heterozygous and homozygous *jak3* mutants for further analysis.

### 3.2. SCID-Related Jak3 Mutation Disrupts Embryonic Lymphopoiesis

**Figure 2:**
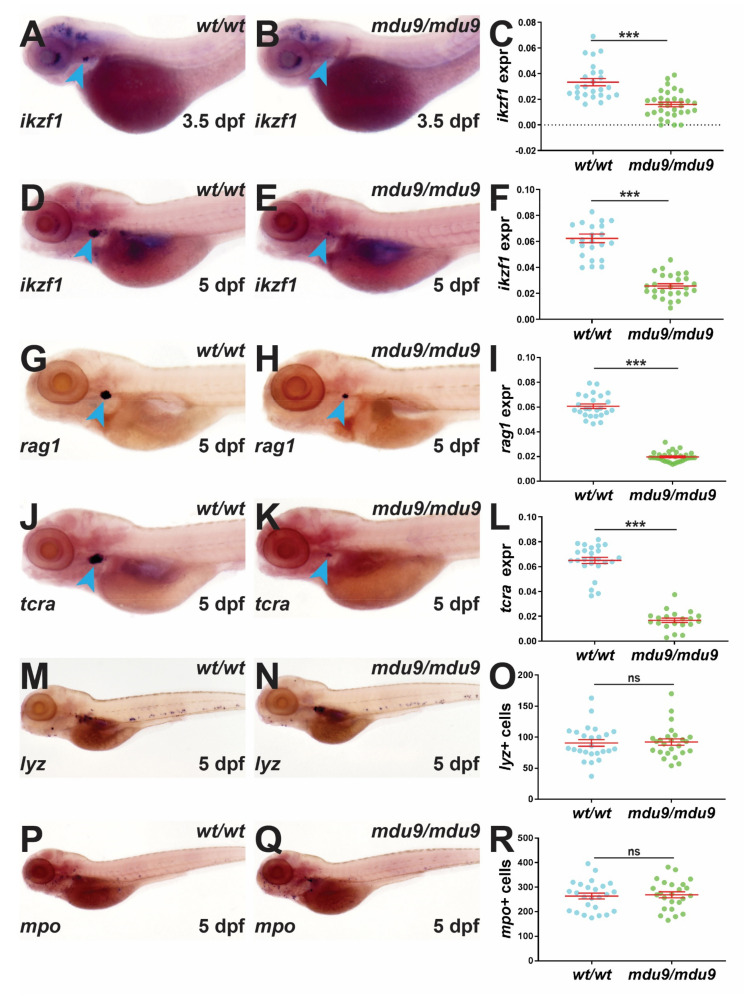
Analysis of embryonic zebrafish carrying Jak3 mutations. Analysis of immune cells in wild-type (*wt*/*wt*) and mutant (*mdu9*/*mdu9*) *jak3* embryos using WISH with *ikzf1* at 3.5 dpf (**A**,**B**) and 5 dpf (**D**,**E**), and *rag1* (**G**,**H**), *tcra* (**J**,**K**), *lyz* (**M**,**N**) and *mpo* (**P**,**Q**) at 5 dpf. Representative embryos are shown with staining in the thymus indicated with blue arrowheads. Individual embryos were assessed for the area of expression for *ikzf1* (**C**,**F**), *rag1* (**I**) and *tcra* (**L**), expressed as a ratio to eye size averaged for individual embryos, or the number of *lyz*+ (**O**) and *mpo*+ (**R**) cells. Results for individuals are shown with the mean and SEM in red and level of statistical significance indicated (*** *p* < 0.001, ns not significant; *n* = 30). This result was confirmed in an additional experiment.

The effect of Jak3 mutations on embryonic hematopoiesis was analyzed using WISH with specific gene markers. Homozygous jak3mdu9/mdu9 mutants showed significantly reduced expression of ikzf1, a marker of early T lymphocyte progenitors in the developing thymus [28], at 3.5 dpf compared to *jak3^wt^*^/*wt*^ siblings (Figure 2A–C). This decrease in thymus staining was sustained at 5 dpf (Figure 2D–F), at which time reduced expression was also observed for *rag1* (Figure 2G–I) and *tcra* (Figure 2J–L), markers of more mature T lymphocytes [29,30]. However, there was no significant difference in the number of cells expressing lyz (Figure 2M–O), a leukocyte marker [31], or *mpo* (Figure 2P–R), a neutrophil marker [32], between jak3wt/wt and jak3mdu9/mdu9 embryos. Expression of rag1 was similarly reduced in homozygous jak3mdu10/mdu10 mutants, with heterozygous jak3wt/mdu10 embryos similar to wild-type siblings (Figure S2A–D), confirming the recessive nature of this mutation.

### 3.3. SCID-Related Jak3 Mutation Perturbs Larval Lymphopoiesis

**Figure 3:**
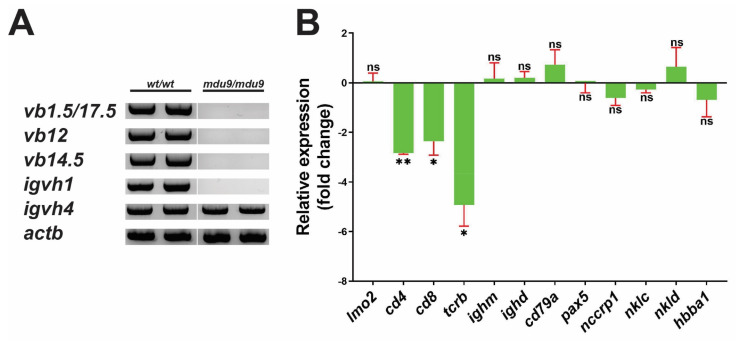
Effect of SCID-derived JAK3 mutations on zebrafish larval lymphopoiesis. Analysis of immune and blood cells in 28 dpf wild-type (*wt*/*wt*) and mutant (*mdu9*/*mdu9*) *jak3* larvae using RT-PCR with primers specific for T cell receptor (TCR) β-chain (*v(d)j-cβ vb1.5*, *vb12*, *vb14.5*) and B cell Ig heavy chain (*igvh1, igvh4*) rearrangements along with *actb* as a control (*n* = 2) (**A**), noting that RT-negative controls yielded no products, or qRT^2^-PCR analysis with the indicated hematopoietic cell markers (**B**). Data is represented as relative fold-change compared to homozygous wild-type (*wt*/*wt*) larvae with mean and SEM shown in red and level of statistical significance of normalized Cq values (relative to control *actb*) indicated (** *p* < 0.01, * *p* < 0.05, ns not significant; *n* = 3).

The effect of Jak3 mutations on zebrafish larval lymphopoiesis was investigated at 28 dpf, when B cell development has commenced [33]. RT-PCR using primers specific for T cell and B cell rearrangements [34,35] showed normal T and B cell rearrangement in wild-type *jak3^wt^*^/*wt*^ individuals, whereas *jak3^mdu9^*^/*mdu9*^ mutants showed no T cell rearrangement and variable B cell rearrangement (Figure 3A). Homozygous *jak3^mdu10^*^/*mdu10*^ larvae displayed a disruption in both T and B cell rearrangements (Figure S2E).

Further analysis was performed using qRT^2^-PCR for a broad range of genes marking HSCs (*lmo2*), T cells (*cd4*, *cd8*, *tcrb*), B cells (*ighm*, *ighd*, *cd79a*, *pax5*), NK cells (*nccrp1*, *nklc*, *nkld*) [36] and red blood cells (RBC) (*hbba1*) [37] (Figure 3B). This revealed a significant and specific reduction in the expression of T cell markers in *jak3^mdu9^*^/*mdu9*^ compared to *jak3^wt^*^/*wt*^ larvae, whereas markers of HSCs, B cells, NK cells and RBC were not significantly altered (Figure 3B).

### 3.4. SCID-Related Jak3 Mutation Perturbs Adult Lymphopoiesis

**Figure 4:**
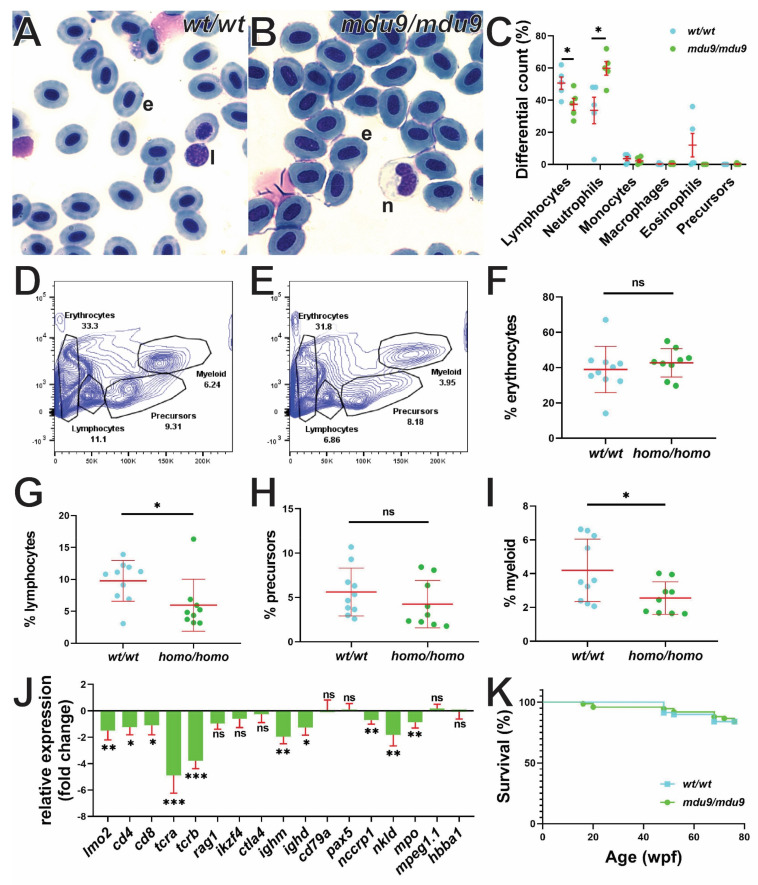
Effect of SCID-derived JAK3 mutations on zebrafish adult hematopoiesis. (**A**–**C**). Analysis of peripheral blood from adult wild-type (*wt*/*wt*) (**A**) and mutant (*mdu9*/*mdu9*) (**B**) *jak3* fish using Giemsa staining (e: erythrocyte; l: lymphocyte; n: neutrophil), including differential counts of the indicated cell populations (**C**). (**D**–**I**) FACS analysis of adult kidney from wild-type (*wt*/*wt*) (**D**) and mutant (**E**) *jak3* fish, along with quantitation of erythrocyte (**F**), lymphocyte (**G**), precursor (**H**) and myeloid (**I**) populations in wild-type (*wt*/*wt*) and mutant (*homo*/*homo*) individuals as indicated. (**J**) Analysis of hematopoietic cell lineages in adult kidney from wild-type and homozygous mutant fish using qRT^2^-PCR analysis with the indicated lineage cell markers. Data is represented as relative fold change compared to wild-type with mean and SEM shown in red and statistical significance of normalized Cq values (relative to control *actb*) indicated (*** *p* < 0.001, ** *p* < 0.01, * *p* < 0.05, ns not significant; *n* = 6). (**K**) Survival analysis of wild-type and mutant *jak3* fish at each week post fertilization (wpf) displayed as a Kaplan–Meier plot (*wt*/*wt*, *n* = 70, *mdu9*/*mdu9*, *n* = 75).

The impact of Jak3 mutations on adult zebrafish lymphopoiesis was first investigated by cytological analysis of peripheral blood. Differential counting revealed a significant reduction in circulating lymphocytes and an increase in neutrophils in *jak3^mdu9^*^/*mdu9*^ compared to *jak3^wt^*^/*wt*^ fish (Figure 4A–C). FACS analysis revealed of adult kidney cells demonstrated a significant decrease in lymphocytes, with myeloid cells also affected (Figure 4D–I). The adult kidney was further analyzed for expression of key cell lineage markers, which revealed significant reduction in the expression of HSC (*lmo2*), T cell (*cd4*, *cd8*, *tcra*, *tcrb*, *rag1*), NK cell (*nccrp1*, *nkld*) and neutrophil (*mpo*) markers in *jak3^mdu9^*^/*mdu9*^ compared to *jak3^wt^*^/*wt*^ adults, with markers of early B cell (*cd79a*, *pax5*), macrophages (*mpeg1.1*) [38] and RBC (*hbba1*) not significantly altered (Figure 4J). Interestingly, expression of mature B cell markers (*ighm*, *ighd*) was significantly reduced in *jak3^mdu9^*^/*mdu9*^ adults. Despite this, Jak3 mutants were generally healthy, with similar survival to wild-type fish (Figure 4K).

### 3.5. SCID-Related Jak3 Mutants Are Susceptible to Lymphocytic Leukemia

**Figure 5:**
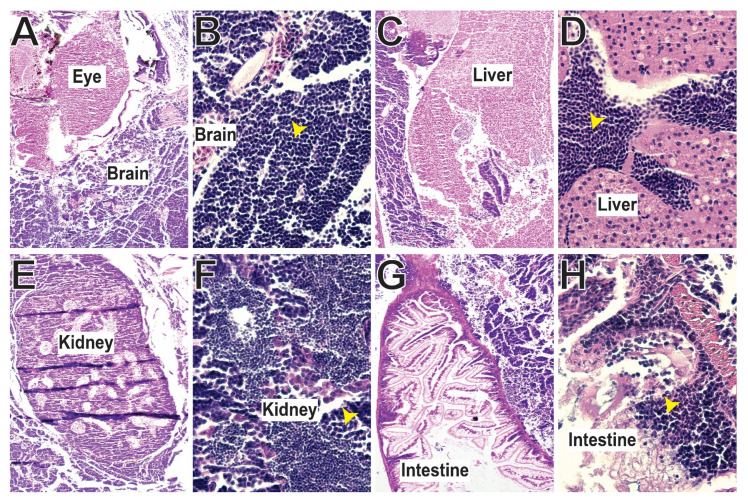
Analysis of lymphocytic leukemia in zebrafish harboring SCID-derived JAK3 mutations. Histology of organs from afflicted zebrafish showing leukemic infiltration into the brain (**A**,**B**), liver (**C**,**D**), kidney (**E**,**F**) and intestine (**G**,**H**) in representative *jak3* mutant fish at 12 months post fertilization imaged at 10× (**A**,**C**,**E**,**G**) and 40× (**B**,**D**,**F**,**H**) magnification. Yellow arrowheads indicate leukemic cell infiltration.

From around 12 months of age Jak3 mutant fish developed visible signs of illness, including skin lesions and tumors affecting around 10% of individuals. Histological analysis revealed invasive, multicentric lymphoid neoplasms, with densely cellular and non-encapsulated neoplastic masses infiltrating the entirety of the fish, variably effacing multiple organs, including the brain (Figure 5A,B), liver (Figure 5C,D), kidney (Figure 5E,F) and intestine (Figure 5G,H). The monomorphic round neoplastic cells were arranged in sheets, supported by minimal fibrovascular stroma, and possessed basophilic cytoplasm with distinct borders and singular ovoid nuclei of stippled chromatin and more than one deeply eosinophilic nucleoli, characteristic of lymphocytic leukemia.

## 4. Discussion

Members of the IL-2R family represent the main cytokine receptors controlling lymphoid development and include the receptors for IL-2, IL-4, IL-7, IL-9, IL-15 and IL-21 [39,40,41,42]. These all share the IL-2Rγc chain that signals via JAK3 and downstream effectors, such as STAT5, PI3K and IRS, to regulate the generation, proliferation and function of various immune cell populations [43]. Disruption of several of these signaling components has been shown to impair normal lymphoid development, with inactivating JAK3 mutations associated with autosomal-recessive SCID in humans and mice [13,14,44,45]. Zebrafish possesses many of the constituent chains of the IL-2R family, including two IL-2Rγc proteins [17] and a single Jak3 with high sequence conservation to its mammalian counterparts [3]. Zebrafish *jak3* was found to be expressed in the thymus during embryogenesis (Figure S1A–E), with higher expression observed in the adult spleen, kidney, and gills (Figure S1E), consistent with the predominantly lymphoid expression of mammalian *JAK3* [46,47,48]. Collectively, this suggested a similar biological role for JAK3 across vertebrates, which encouraged us to generate a zebrafish SCID model based on an appropriate *jak3* mutant. SCID-causing JAK3 mutations are found throughout the JAK3 protein, [13,27] such as a non-sense mutations in the PKD [15] and frame-shift mutations in the SH2 [15], FERM [49] or kinase [50] domains. Mis-sense mutations have also been identified, including an E481G substitution mutation in the SH2 domain leading to a milder SCID phenotype [50] and a compound PKD C759R and SH2 domain non-sense mutation [50]. In this study the PKD of zebrafish Jak3 was targeted, with two independent mutant alleles identified each truncating at the same amino acid within this domain.

Human SCID caused by JAK3 mutation is characterized by severely reduced T and NK cells, but with B cells present, although with compromised functionality [14,15]. The zebrafish *jak3* mutants displayed a severe reduction in T lymphocytes during embryonic hematopoiesis that continued into the larval stage. NK cells were reduced in the adult, with evidence of abrogated B cell maturation and some disruption of the neutrophil compartment. This is consistent with a study showing *jak3* mutants have reduced early T cells but where the effects on B cells and NK cells or into adulthood were not characterized [20], and another solely reporting depletion of T and NK cells in the adult kidney [19]. The data presented here for the first time characterized the complete impact of zebrafish *jak3*-deficiency throughout the life-course.

The phenotype of human SCID due to JAK3 mutations is very similar to that observed in X-linked SCID (X-SCID) resulting from mutations in the IL2Rγc-chain [51]. Both Jak3 and Il-2rγc knockout mice also developed a form of SCID, although in this case with markedly reduced T, NK and B cells [44,45,52,53,54]. Inactivating mutations in zebrafish Il2rγc.a resulted in a SCID phenotype, with reduced T and NK cells but normal B cells [18,55], like in humans. The variation in B cell numbers between mouse and human mutants has been attributed to IL-7 being a pre-B cell growth factor in mice but not in humans [56]. Patients harboring mutations in IL-7 receptor alpha chain have a T-B+NK+ SCID phenotype [56,57]. Mice with IL-7 and IL-7R deficiency possessed reduced levels of T and B cells [58,59], with IL-7R deficient mice having normal NK cell levels [60]. Zebrafish carrying an inactivating mutation of Il-7 displayed reduced T cells, but unaffected B cells again similar to humans [20], with NK cells not characterized in that study. The difference between zebrafish Jak3 and Il2rγc.a mutants might be due to the duplicated Il2rγc.b receptor in zebrafish [17], which may play a role in B cell development and maturation.

The functional role of JAK3 in lymphopoiesis is well characterized. However, its role in myeloid cell development and differentiation is less explored. One publication has identified impaired myelopoiesis in Jak3-deficient mice, with the maturity of both neutrophils and monocytes impacted [61]. Another identified *JAK3* as a primary response gene induced by granulocyte colony-stimulating factor (G-CSF) with overexpression of JAK3 associated with cell growth inhibition and terminal granulocytic differentiation [62]. This study has revealed disruption of neutrophil homeostasis in zebrafish *jak3* mutants, being decreased in the kidney and increased in peripheral blood. Delineating the mechanistic details remains a worthwhile undertaking.

Notably, the zebrafish *jak3* mutants survived to adulthood, which contrasts with the poor prognosis in human JAK3-SCID, with patients succumbing to the disease during early childhood if left untreated [63]. This probably reflects the greater dependence on innate compared to adaptive immunity in teleost fish, with adaptive immunity not established until 4 weeks of age [64], with several higher level aspects not present [65]. However, adult zebrafish *jak3* mutants developed a malignant lymphoid leukemia, which invaded multiple organs like brain, kidney, liver and intestine. We were unable to definitively characterize this due to a lack of key reagents, but this observation is consistent with primary immune deficiency patients developing cancer, particularly hematological rather than solid tumors [66]. This suggests that the immunodeficiency caused by JAK3 mutation significantly impacts tumor immunity.

## 5. Conclusions

This study describes the generation and characterization of a zebrafish model of SCID due to Jak3 deficiency. These zebrafish mutants showed depletion of embryonic, larval and adult T cells, reduced B cell maturation and NK cell numbers in adults, which also displayed disrupted neutrophil homeostasis and susceptibility to lymphoid malignancy. It is anticipated that this line will enable further exploration of the role of Jak3 in the myeloid lineage, as well as enable studies examining the microbiota and facilitate xenotransplantation studies, as recently described for an IL-2Rγc model of SCID [18].

## Data Availability

All data generated or analyzed during this study are included in this published article (and its Supplementary Materials).

## Supplementary Materials

The following supporting information can be downloaded at: https://www.mdpi.com/article/10.3390/biom12101521/s1, Figure S1: Expression of zebrafish *jak3* during embryogenesis and in adult tissues, Figure S2: Phenotypic analysis of zebrafish carrying alternate Jak3 allele.
