# Supplementary material for: Zebrafish Model of Severe Combined Immunodeficiency (SCID) Due to JAK3 Mutation
Source: Biomolecules. 2022 Oct 20;12(10):1521. doi: 10.3390/biom12101521 (PMC9599616; doi:10.3390/biom12101521)
Supplement: Supplementary file 1 [file biomolecules-12-01521-s001.zip › biomolecules-1945281-supplementary.pdf]

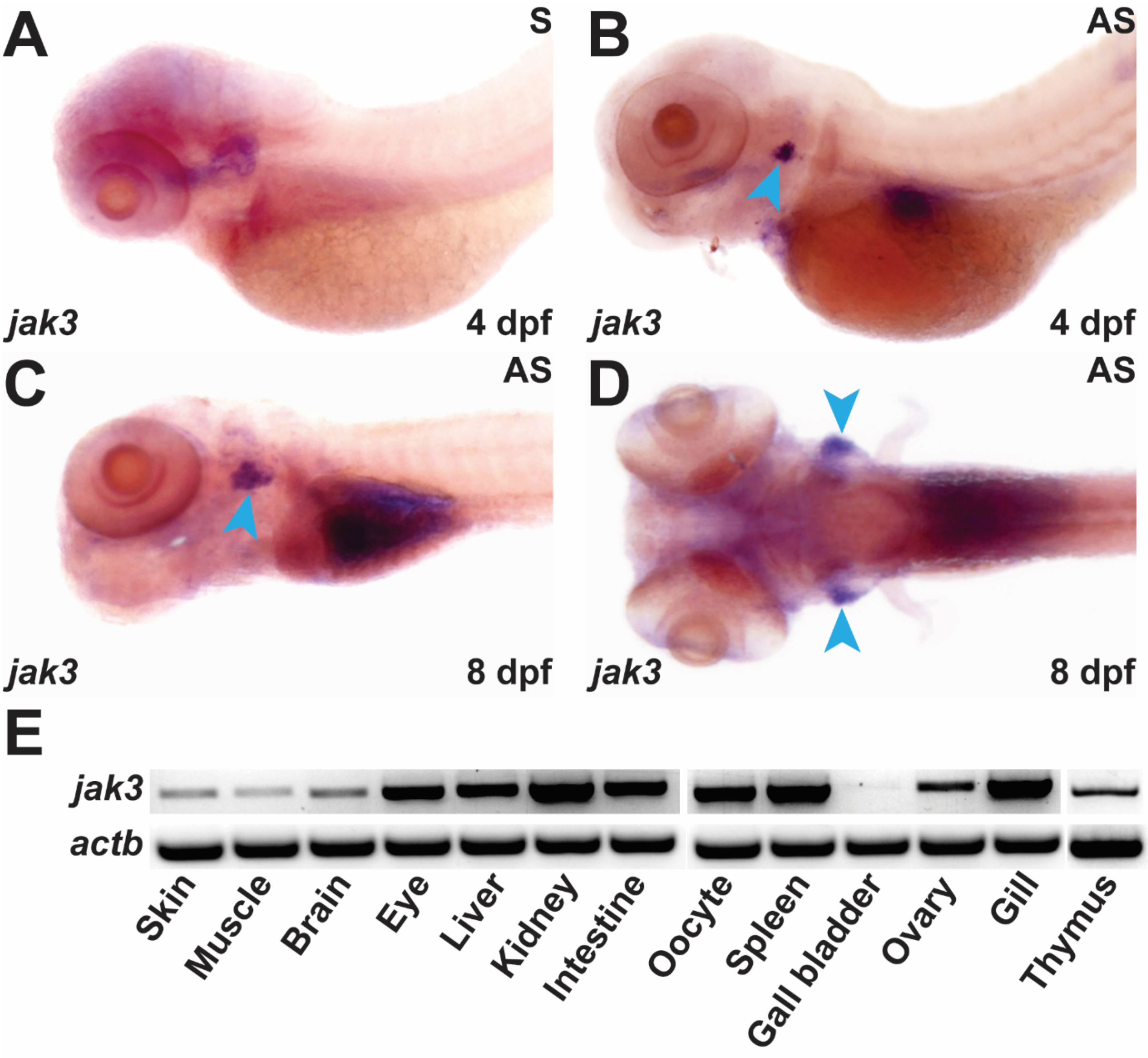

Figure S1: Expression of zebrafish *jak3* during embryogenesis and in adult tissues.

A-E. Expression of *jak3* in wild-type embryos subjected to WISH with sense (S) and anti-sense (AS) *jak3* probes at the indicated time points. Staining in the thymus is indicated with blue arrow heads. Representative embryos are shown as lateral (A-C), or dorsal (D) view, with anterior to the left (n=30). E. Expression of *jak3* in adult tissues using RT-PCR analysis of total RNA extracted from the indicated tissues with *jak3* primers, along with *actb* as a loading control. RT-negative controls yielded no products (data not shown).

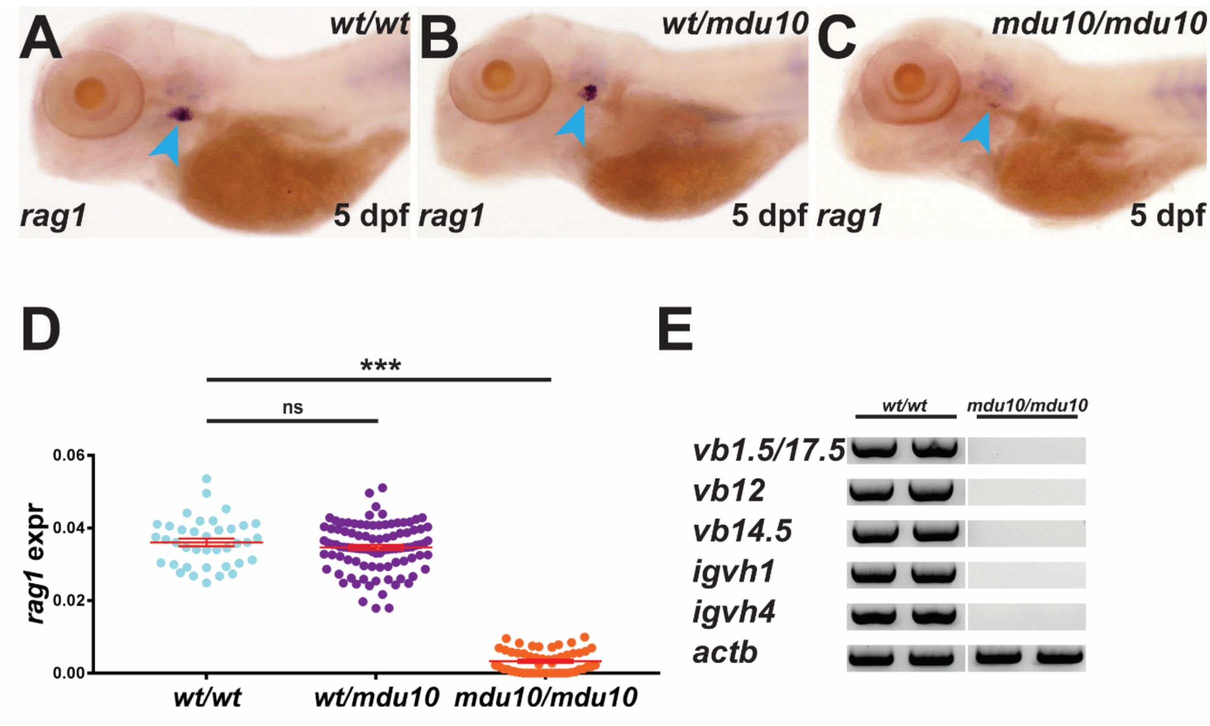

Figure S2: Phenotypic analysis of zebrafish carrying alternate JAK3 allele.

A-D. Analysis of T lymphocytes in wild-type (*wt/wt*), heterozygous (*wt/mdu10*) and homozygous (*mdu10/mdu10*) mutant *jak3* embryos using WISH with *rag1* at 5 dpf with representative embryos shown (A-C). Individual embryos were assessed for the area of expression for *rag1,* assessed as a ratio to eye size averaged for each embryo (D), with the mean and SEM shown in red and level of statistical significance indicated (*** $p<0.001$, ns not significant; n>30). E. Analysis of T and B lymphocytes in 28 dpf wild-type (*wt/wt*) and homozygous (*mdu10/mdu10*) mutant *jak3* juvenile, using RT-PCR with primers specific for T cell receptor (TCR) β-chain (v(*d*)*j-cβ vb1.5*, *vb12, vb14.5*) and B cell Ig heavy chain (*igvh1, igvh4*) rearrangements and *actb* as a control (n=2). RT-negative controls yielded no products (data not shown).
